# Moderate alcohol consumption does not impair overload-induced muscle hypertrophy and protein synthesis

**DOI:** 10.14814/phy2.12333

**Published:** 2015-03-16

**Authors:** Jennifer L Steiner, Bradley S Gordon, Charles H Lang

**Affiliations:** Department of Cellular and Molecular Physiology, Penn State College of MedicineHershey, Pennsylvania

**Keywords:** Ethanol, hypertrophy, mTORC1, overload, protein synthesis

## Abstract

Chronic alcohol consumption leads to muscle weakness and atrophy in part by suppressing protein synthesis and mTORC1-mediated signaling. However, it is unknown whether moderate alcohol consumption also prevents overload-induced muscle growth and related anabolic signaling. Hypertrophy of the plantaris muscle was induced by removal of a section of the gastrocnemius and soleus muscles from one leg of C57BL/6 adult male mice while the contralateral leg remained intact as the sham control. A nutritionally complete alcohol-containing liquid diet (EtOH) or isocaloric, alcohol-free liquid diet (Con) was provided for 14 days post-surgery. EtOH intake was increased progressively (day 1–5) before being maintained at ∽20 g/day/kg BW. The plantaris muscle from the sham and OL leg was removed after 14 days at which time there was no difference in body weight between Con and EtOH-fed mice. OL increased muscle weight (90%) and protein synthesis (125%) in both Con and EtOH mice. The overload-induced increase in mTOR (Ser2448), 4E-BP1 (Thr37/46), S6K1 (Thr389), rpS6 (Ser240/244), and eEF2 (Thr56) were comparable in muscle from Con and EtOH mice. Modulation of signaling upstream of mTORC1 including REDD1 protein expression, Akt (Thr308), PRAS40 (Thr246), and ERK (Thr202/Tyr204) also did not differ between Con and EtOH mice. Markers of autophagy (ULK1, p62, and LC3) suggested inhibition of autophagy with overload and activation with alcohol feeding. These data show that moderate alcohol consumption does not impair muscle growth, and therefore imply that resistance exercise may be an effective therapeutic modality for alcoholic-related muscle disease.

## Introduction

Skeletal muscle disease occurs in 40–60% of chronic alcoholics, and is therefore more prevalent than many other myopathies in addition to being of greater incidence than alcohol-induced cirrhosis (Martin et al. 1985; Estruch et al. 1993). Past work related to alcohol-induced skeletal muscle disease has primarily focused on determining the mechanism(s) through which alcohol decreases skeletal muscle size and function; while to our knowledge, no reports exist investigating whether alcohol also hinders muscle growth. This information would prove important to the treatment of alcohol-induced muscle disease as interventional therapies to prevent, offset or reverse the loss of muscle mass, and strength resulting from prolonged alcohol intake are lacking.

Muscle mass is regulated by the balance (or lack thereof) between rates of protein synthesis and protein breakdown. Both acute and chronic alcohol consumption inhibits synthesis to a greater extent than degradation (Lang et al. 2001). Therefore, much of the current knowledge has focused on elucidating the effects of alcohol on the canonical mammalian target of rapamycin (mTOR) signaling pathway as it represents the predominant metabolic pathway in the control of protein synthesis. Following detection of an anabolic signal, upstream stress and energy sensors like Regulated in Development and DNA Damage responses-1 (REDD1), AMP-activated protein kinase (AMPK), and the PI3K/Akt pathway modulate Rheb-GTP loading via Tuberous Sclerosis Complex 1/2 (TSC1/2) inhibition leading to mTOR activation at the lysosomal membrane (Wang and Proud 2006; Zheng et al. 2014). S6K1 and eukaryotic initiation factor 4E- binding protein-1 (4E-BP1) are two of the primary substrates downstream of mTOR complex-1 (mTORC1) and their phosphorylation enhances initiation of protein translation and elongation, ultimately resulting in stimulation of muscle protein synthesis following various anabolic stimuli such as resistance exercise.

Initiation of mRNA translation and protein synthesis is impaired by acute and chronic alcohol intake likely via suppression of mTORC1 signaling to its downstream substrates 4E-BP1 and S6K1 (Lang et al. 2004, 2007, 2009). S6K1 also phosphorylates eukaryotic elongation factor -2 (eEF2) kinase which decreases eEF2 phosphorylation and slows elongation of peptide chains at the ribosome (Wang et al. 2001; Browne and Proud 2002). Acute alcohol intoxication in mice, as well as in vitro exposure of C2C12 myocytes to alcohol, suppresses elongation (assessed by an increase in eEF2 (Thr^56^) phosphorylation) in addition to translation initiation (Hong-Brown et al. 2007; Steiner and Lang 2015).

Along with these alcohol-induced changes observed under basal conditions, the protein synthetic response to acute anabolic stimuli including growth factors (insulin), nutrients (feeding and the amino acid leucine), and electrically stimulated muscle contraction is also impaired by acute alcohol intoxication (Kumar et al. 2002; Lang et al. 2003; Sneddon et al. 2003; Steiner and Lang 2014). Therefore, it has typically been inferred that alcohol would prevent stimulation of muscle growth in response to an anabolic stimulus; however, this has not yet been directly examined. Thus, in the current study we tested the hypothesis that moderate alcohol consumption impairs muscle hypertrophy induced by mechanical overload.

## Methods

### Animals

Male viral antibody free C57BL/6 mice aged 11–13weeks  were purchased from Charles River Laboratories (Wilmington, MA) and acclimated for at least 1 week to the animal facility at the College of Medicine at Penn State Hershey prior to experimental use. Mice were housed (2/cage) in shoe-box cages with corn cob bedding under controlled environmental conditions (12:12 light:dark), and were provided Teklad Global 2019 (Harlan Teklad, Boston, MA) and water ad libitum until the start of the experiment. All experimental procedures were performed in accordance with the National Institutes of Health guidelines for the use of experimental animals and were approved by the Institutional Animal Care and Use Committee of The Pennsylvania State University College of Medicine.

### Experimental design

All mice were individually housed and acclimated to the control (alcohol-free) Lieber-DeCarli ‘82 Shake and Pour rodent liquid diet (product # F1259SP; Bio-Serv, Flemington, NJ) for 1 week prior to initiation of muscle overload. Body composition (fat and lean mass) was assessed noninvasively on conscious animals using a ^1^H-NMR analyzer (Bruker LF90 Proton-NMR Minispec: Bruker Optics, Woodlands, TX) immediately prior to muscle overload and prior to sacrifice 2 weeks later. To stimulate muscle growth, unilateral functional overload of the plantaris muscle was performed on all mice. The unilateral model of overload was chosen as it leads to similar augmentation of mTORC1 signaling and muscle hypertrophy as that reported for bilateral overload (Bodine et al. 2001; McGee et al. 2008; Pérez-Schindler et al. 2013) and would allow for exposure of the muscle to identical levels of alcohol and circulation factors (hormones, nutrients, etc.).

To induce overload of the plantaris mice were first anesthetized with 3% inhaled isoflurane maintained via a nose cone on a heated surface. Under aseptic conditions both hind legs were shaved and a small incision was made on the lower leg exposing the Achilles tendon and ankle extensor muscles. More than half of the gastrocnemius and soleus muscle was removed without disturbing the plantaris nor its neural-vascular supply using standard methods (Bodine et al. 2001; Bodine and Baar 2012). Following irrigation with sterile saline, 7-0 surgical sutures (Ethicon, Cat # 1647G) were used to close the wound. An identical procedure was performed on the contralateral leg except that the tendons were not cut and the soleus and gastrocnemius remained intact to serve at the sham control. Mice were administered analgesic (burprenorphine, Reckitt Benckiser Pharmaceuticals Inc. Richmond, VA; 0.1 mg/kg in sterile saline) subcutaneously and then allowed to recover before being returned to individual cages. Approximately 6 h later, a second dose of buprenorphine was given subcutaneously to all animals. All animals were monitored for signs of sickness or pain and wounds were checked daily for signs of infection. All mice survived the surgery and no signs of infection or distress were observed. Cage activity was also monitored each day and no preference for ambulation on the nonoverloaded leg was observed.

Following surgery, animals were either acclimated to the alcohol-containing liquid diet (product # F1258SP; Bio-Serv, Flemington, NJ) or continued consuming the control liquid diet. Animals were pair-fed (i.e., controls received the amount of food consumed by the EtOH mice the day before) to match any potential differences in feeding behavior caused by the addition of the alcohol to the diet. Food consumption was assessed daily and fresh food was provided daily. The alcohol treatment group (EtOH, *n* = 10), was acclimated to the alcohol containing diet using a ramping protocol which included 1 day of 10%, and 2 days each of 16%, 22%, and 30% kcal from alcohol. Mice then received the complete 36% Lieber-DeCarli alcohol diet formulation for 3 days before the concentration was lowered back to 30% to prevent a further decline in food consumption and body weight as has been previously reported (Freund 1970). The 14-day treatment period was selected as prior investigations reported significant increases in plantaris weight as well as protein synthesis at this time point (Bodine et al. 2001; Pérez-Schindler et al. 2013; Baehr et al. 2014). On day 14 post-mechanical overload animals were weighed and body composition was measured using the ^1^H-NMR analyzer before they were deeply anesthetized via isoflurane inhalation (4–5%) and the plantaris muscle (tendon to tendon) was carefully removed intact from both legs. Muscles were immediately frozen in liquid nitrogen and stored at −80°C until analysis.

### Protein synthesis

In vivo protein synthesis was assessed using the SUnSET method and an antibody against puromycin (Kerafast, Boston, MA) (Goodman et al. 2011; Goodman and Hornberger 2013). All animals were administered an intraperitoneal injection of 0.04 *μ*mol/g body weight of puromycin dissolved in sterile saline 30 min prior to the removal of the plantaris muscles. Western blotting procedures were performed to visualize puromycin incorporation (Kelleher et al. 2013).

### Western blotting

The plantaris was weighed and immediately homogenized in ice-cold buffer containing (in mM): 20 HEPES (pH 7.4), 2 EGTA, 0.2 EDTA, 100 KCl, 50 *β*-glycerophosphate, 50 NaF, 0.5 sodium orthovanadate, 1 Benzamidine, 0.1 PMSF, 1 DTT. Samples were centrifuged at 10,000 *g* for 10 min and the supernatant was used in subsequent analyses. Protein concentration was quantified using Bio-Rad Protein Assay Dye reagent concentrate (Hercules, CA) and SDS-PAGE was performed using equal amounts of total protein per sample and Criterion 4-20% gradient gels (Bio-Rad, Hercules, CA). Following Ponceau S (Aqua Solutions, Deer Park, TX) staining to verify loading of equal amounts of protein, PVDF membranes were blocked in 5% nonfat dry milk, and then incubated overnight at 4°C with primary antibody. Antibodies included (Cell Signaling, Beverly, MA, unless otherwise noted): S6K1, S6K1 (Thr^389^), rpS6, rpS6 (Ser^240/244^), 4E-BP1 (Bethyl Laboratories, Montgomery, TX), 4E-BP1 (Thr^37/46^), eEF2, eEF2 (Thr^56^), ERK1/2 (Thr^202^/Tyr^204^), p42/44 MAPK, mTOR, mTOR (Ser^2448^), REDD1 (ProteinTech, Chicago, IL), Akt, Akt (Thr^308^), PRAS40, PRAS40 (Thr^246^), ULK1, ULK1 (Ser^757^), p62, LC3A/B, and GAPDH. The appropriate secondary antibodies were added in 5% nonfat milk for 1–2 h prior to washing with TBST. Images were visualized on the FluorChem M Multifluor System (ProteinSimple, San Jose, CA) following exposure to Pierce ECL Western blotting substrate (Thermo Scientific, Waltham, MA). Images were analyzed using AlphaView (ProteinSimple) and Image J software (NIH).

### Statistics

Data were analyzed using commercially available statistical software (GraphPad, San Diego, CA and SigmaPlot, Systat, San Jose, CA). For assessment of changes across the four treatment groups (Control-Sham; Control-OL; EtOH-Sham; EtOH-OL), a repeated measures two-way ANOVA (overload x alcohol) with Student–Neuman–Keuls post hoc tests was performed. Data are presented as mean ± SEM and considered significant when *P* < 0.05.

## Results

Body weight assessed prior to muscle overload did not differ between groups (Con: 24.7 ± 0.2 g; EtOH: 24.8 ± 0.2 g). Fat and lean mass also did not differ between groups (data not shown). After 14 days of mechanical overload, body weight immediately prior to sacrifice remained comparable between groups (Con: 25.9 ± 0.4 g; EtOH: 24.8 ± 0.7 g), as did fat and lean mass (data not shown), supporting the efficacy of the pair-feeding regime.

**Figure 1:**
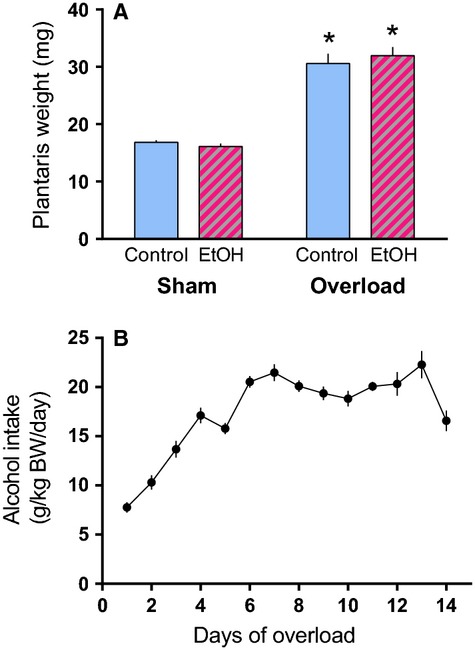
Muscle overload for 14 days increases plantaris weight despite alcohol consumption. (A) Weight of the plantaris in the control (Sham) and contralateral overloaded (Overload) leg after 14 days of either control or alcohol (EtOH) treatment (*n* = 10/group). (B) Amount of alcohol consumed daily (*n* = 10) during the 14-day overload period. *P < 0.05, indicates statistical difference from the corresponding Sham condition. Values are expressed as means ± SE.

**Figure 2:**
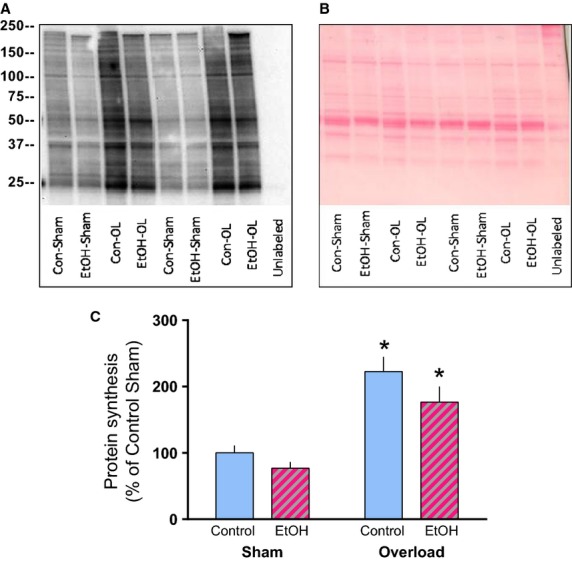
Overload increases protein synthesis in control and alcohol-fed mice. Puromycin was injected 30 min prior to tissue collection and a representative image of the Western blot (A) and the corresponding Ponceau S stained membrane used to verify loading (B) is shown. Treatment groups are labeled beneath each lane (Control, *n* = 10; EtOH, *n* = 10). (B) Quantification of the Western blot image expressed as a percentage of the Control-Sham condition. *P < 0.05, indicates statistical difference from the corresponding Sham condition. Values are expressed as means ± SE.

Following 14 days of mechanical overload the absolute weight of the plantaris increased the same extent in control and alcohol-fed mice when expressed in mg (Fig.1A) or normalized to body weight (data not shown). The increase in muscle mass (OL vs. sham muscle within an animal) over the 14-day period did not differ between control (91.3 ± 8.1%) and alcohol-fed mice (92.1 ± 7.3%). Food consumption was monitored to ensure changes in muscle mass were not due to dietary differences and that the EtOH mice had in fact ingested alcohol. Accordingly, over the first 7 days alcohol intake increased progressively so that by days 7–14 EtOH mice were consuming approximately 18–20 g of alcohol per kg body weight per day (Fig.1B). Mice were given ad libitum access to food until sacrifice resulting in an average blood alcohol concentration of 8.6 ± 2.8 mmol/L at the time of tissue collection. Finally, analogous to muscle weight, the increase in protein synthesis in the plantaris muscle did not differ between control (125.2 ± 11.1%) and alcohol-treated mice (127.4 ± 12.8%) following 14 days of overload (Fig.2).

**Figure 3:**
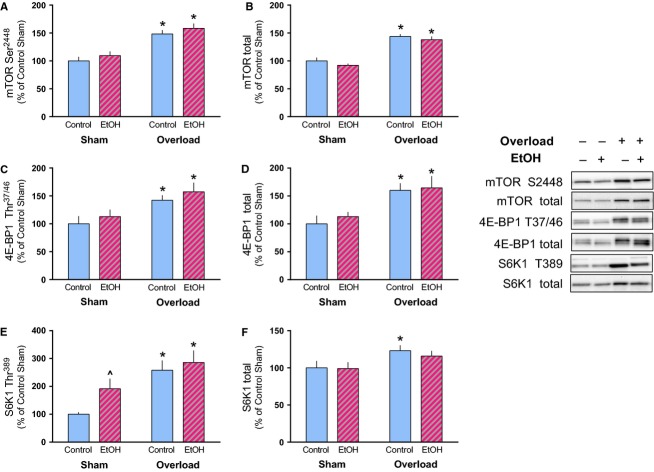
Alcohol does not impair mTORC1 signaling induced by muscle overload. The relative amount of phosphorylated and total mTOR (A, B), 4E-BP1 (C, D) and S6K1 (E, F) were measured in the plantaris muscle after 14 days of muscle overload. Representative images of each marker correspond to the sample order depicted in the graphs. *P < 0.05, indicates differences from Sham condition within that treatment while ^ indicates a trend for differences from control-sham, *P* = 0.06. Values are expressed as means ± SE.

**Figure 4:**
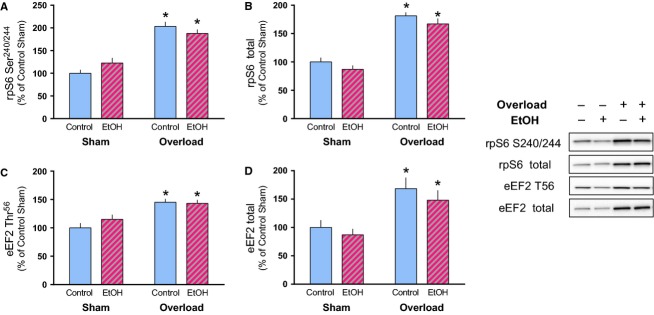
Muscle overload-induced increases in S6K1 substrates rpS6 and eEF2 are not prevented by alcohol consumption. The relative amount of phosphorylated and total rpS6 (A, B) and eEF2 (C, D) were measured in the plantaris muscle after 14 days of overload. Representative images of each marker correspond to the sample order depicted in the graph. *P < 0.05, indicates difference from Sham condition within that treatment. Values are expressed as means ± SE.

As alcohol can suppress the stimulation of mTORC1 activity and protein synthesis in skeletal muscle (Kumar et al. 2002; Lang et al. 2003; Steiner and Lang 2014), mTOR phosphorylation and phosphorylation of its substrates were assessed. Mechanical overload increased the phosphorylated and total amount of mTOR (Ser2448) and its substrate 4E-BP1 (Thr37/46) similarly in Control and EtOH mice (Fig.3A–D). Phosphorylation of S6K1 (Thr389) was also increased in both groups by overload but total S6K1 was only increased in Control mice following overload (Fig.3E and F). Consumption of the alcohol-containing diet did not impair the overload-induced increase in rpS6 (Ser240/244), eEF2 (Thr56) or their respective total amounts (Fig.4A–D).

**Figure 5:**
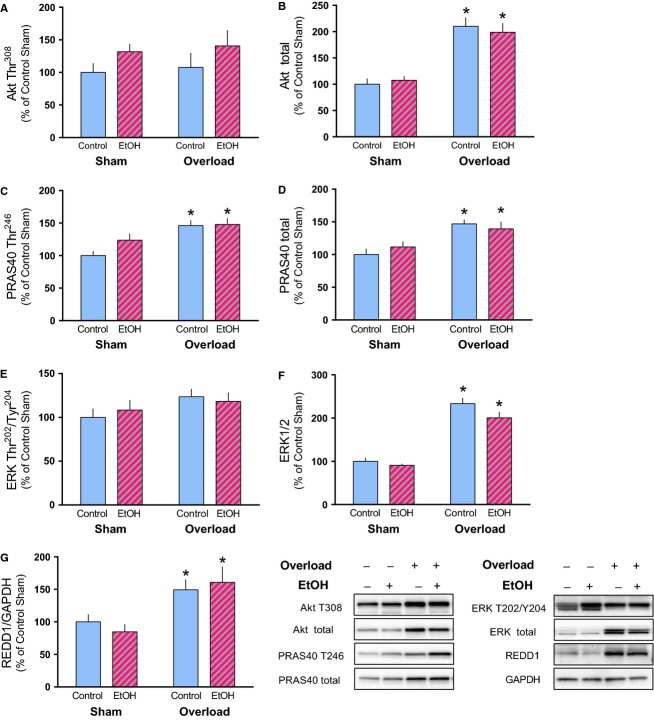
Expression of proteins upstream of mTORC1 was not differentially altered by alcohol in response to muscle overload. The relative amount of phosphorylated and total Akt (A, B), PRAS40 (C, D), ERK (E, F), and REDD1 (G) were measured in the plantaris muscle after 14 days of muscle overload. Representative images are shown corresponding to the sample order presented graphically. *P < 0.05, indicates significant difference from Sham condition within that treatment. Values are expressed as means ± SE.

Several proteins and pathways impact mTORC1 signaling and protein synthesis in response to a variety of metabolic perturbations. Activation of the IRS1/PI3K pathway, while not required, may also contribute to overload-induced muscle growth (Bodine et al. 2001; Spangenburg et al. 2008). Muscle overload did not alter the phosphorylation of Akt (Thr308) despite a ∽100% increase in the total amount of Akt in both Con and EtOH groups (Fig.5A and B). PRAS40 is phosphorylated by Akt at Thr246 relieving its inhibition on mTORC1 (Wang et al. 2012). Muscle overload increased both phosphorylated and total amounts of PRAS40 equally in control and EtOH (Fig.5C and D). MAPK/ERK signaling has been implicated in protein synthesis in response to anabolic stimuli and may have both mTOR-dependent and -independent actions (Widegren et al. 2001; Wang and Proud 2006; Roux et al. 2007; Anjum and Blenis 2008). Muscle overload in both treatment groups (Con and EtOH) increased total ERK expression, while no concomitant increase in its phosphorylation on Thr202/Tyr204 was detected (Fig.5E and F). Finally, the negative regulator of mTORC1 activity, REDD1 was increased by muscle overload similarly in control and EtOH mice (Fig.5G). As there was no corresponding total protein to control for loading of REDD1, it was expressed relative to GAPDH as we found that mechanical overload increased the amount of other common loading controls including actin, *β*-tubulin, and eIF4E (data not shown).

**Figure 6:**
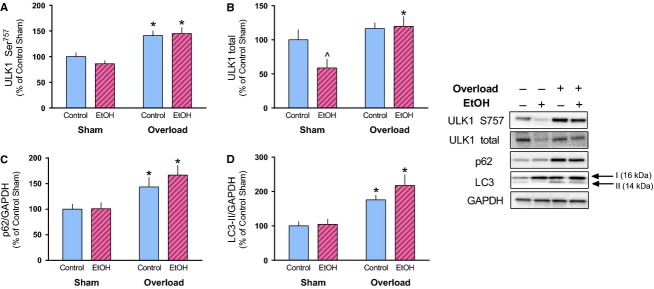
Markers of autophagy were differentially regulated by alcohol in response to muscle overload. The relative amount of phosphorylated and total ULK1 (A, B), total p62 (C), and LC3-II (D) were determined in the plantaris muscle 14-days after muscle overload. Representative images of each marker are provided along with a representative image for GAPDH which was used for normalization of p62 and LC3A/B-II expression. *P < 0.05, indicates difference from Sham condition within that treatment; ^*P* < 0.05, indicates difference from Control-Sham. Values are expressed as means ± SE.

Signaling through mTORC1 may also regulate muscle mass via autophagy, which alcohol antagonizes under basal conditions (Kim et al. 2011; Thapaliya et al. 2014). Presently, alcohol decreased the total amount of ULK1 protein in the sham leg and tended to decrease its phosphorylation (Ser757) as well (Fig.6A and B). However, alcohol had no impact on the overload-induced increase in both total ULK1 and phosphorylation on Ser757. As p62 is degraded during autophagy its accumulation may be indicative of suppression or inhibition of autophagy (Pankiv et al. 2007). Muscle overload increased p62 50–60% in control and alcohol-fed mice (Fig.6C). Finally, LC3A/B-II showed a significant increase following overload irrespective of the treatment condition (Fig.6D).

## Discussion

Evidence supports an alcohol-mediated loss of muscle mass and function; conversely, no investigations have tested whether alcohol reduces skeletal muscle growth induced by an anabolic stimulus despite its direct clinical relevance (Martin and Peters 1985). Consistent with previous work in this model, overload of the plantaris muscle led to significant hypertrophy concomitant with a sustained increase in protein synthesis at 14 days (Bodine et al. 2001; Miyazaki et al. 2011; Pérez-Schindler et al. 2013; Baehr et al. 2014). Moderate consumption of alcohol did not prevent this growth nor did it antagonize the rate of protein synthesis and mTORC1 signaling increased by overload. Conversely, data suggest that alcohol enhances autophagy in the overloaded muscle while it appears to be decreased in the Control group. Despite the differential modulation of autophagic signaling following overload, moderate alcohol consumption did not impair muscle growth as was originally hypothesized.

It is accepted that mTORC1 is central in the control of muscle protein synthesis and accordingly, modulation of this pathway has been well characterized in response to acute and chronic alcohol consumption (Lang et al. 2003, 2007, 2009; Korzick et al. 2013). Both acute and chronic alcohol intoxication antagonize translational efficiency and impair protein synthesis in association with decreased mTOR, 4E-BP1, S6K1, and rpS6 phosphorylation within skeletal muscle of mice and rats (Lang et al. 2004, 2007, 2009). However, in the present investigation comparison between the sham conditions (control vs. alcohol) provided no evidence of a main effect of alcohol with the exception of a reduction in the total amount of ULK1. It is likely that the absence of alcohol-related changes was either the result of the low blood alcohol concentration at the time of tissue collection (<10 mmol/L) (compared to models of acute intoxication) or the relatively short duration of the alcohol feeding (2 weeks vs. >12 weeks) compared to that in other chronic models (Korzick et al. 2013).

In addition to the generalized lack of basal changes in alcohol-treated muscle, the current findings also show that alcohol did not antagonize the induction of mTOR activity and protein synthesis during a constant anabolic stimulus which is in contrast to our previous work (Kumar et al. 2002; Lang et al. 2003; Vargas and Lang 2008; Steiner and Lang 2014). For example, twice daily gavage of alcohol (50 mmol/kg BW) in rats impaired muscle regrowth and suppressed rpS6 and 4E-BP1 phosphorylation in the gastrocnemius when it was reloaded for 3 days following a period of immobilization (Vargas and Lang 2008). Further, acute alcohol intoxication immediately prior to treatment with anabolic agents like stimulated muscle contraction, insulin or leucine, also antagonized the anabolic response (Kumar et al. 2002; Lang et al. 2003; Steiner and Lang 2014). The lack of changes observed presently may be related to either the timing of the alcohol (and nutrient) intake in relation to tissue collection; the lower, more persistent level of alcohol versus a single higher level spike just prior to the stimulus; or the prolonged nature of the current anabolic event compared with the shorter more acute stimulation previously tested.

Muscle growth occurs when the rate of protein accumulation exceeds the rate of protein degradation and while alcohol appears to have little effect on proteasome-mediated degradation, it does increase markers of autophagy to potentially contribute to the atrophic phenotype (Thapaliya et al. 2014). Currently, discordant changes in markers of autophagy were observed with the combination of overload and alcohol feeding. In control animals, the increase in ULK1 phosphorylation and p62 expression following overload suggests inhibition of autophagy. However, increases in LC3-II could be interpreted as either increased autophagosome synthesis or decreased autophagosome turnover. Therefore, it appears that overload may suppress autophagy which in turn aids muscle growth. In contrast, alcohol decreased overload-induced phosphorylation of ULK1 concomitant with increased LC3-II, suggesting an increase in autophagy. To the best of our knowledge, the autophagic response to overload-induced muscle growth has not been previously characterized, although it is reportedly decreased following an acute bout of resistance exercise (Fry et al. 2013). Loss of autophagy via deletion of Atg7 which is required for LC3-II conjugation, causes severe muscle loss under normal and atrophic conditions, while conversely, constitutive autophagy also worsens muscle wasting (Mammucari et al. 2007; Zhao et al. 2007; Masiero et al. 2009). Therefore, maintenance of an appropriate autophagic balance is essential to the regulation of muscle mass.

The current alcohol feeding paradigm is applicable to individuals consuming moderate amounts of alcohol; however, it is not as representative of situations in which large amounts of alcohol are consumed less frequently (i.e., binge drinking). Replication of the later condition was not feasible due to methodological limitations despite its applicability and interest to a large percentage of the population (Kanny et al. 2013). For instance, administering large boluses of alcohol daily via oral gavage led to poor health in the mice and repetitive intraperitoneal injections could cause organ tissue damage in addition to lacking translational relevance. Therefore, dietary incorporation was the least stressful method of alcohol administration over the 14-day period. Nevertheless, limitations exist including that the dose of alcohol was dependent on the animals' feeding behavior; the alcohol content of the diet had to be progressively increased over the first 5 days, coinciding with the period of time during which larger changes in signaling events may have been occurring; and lastly, that there were likely to be differences in when, and in what sized portions each animal consumed the total volume of food (i.e., all at once or more slowly over the course of the 24-h period) which would thereby impact blood alcohol levels. However, previous reports show that the plasma alcohol concentration is highest in mice 2–3 h after the start of the dark cycle and remain elevated until the beginning of the light cycle (Jelic et al. 1998; Filiano et al. 2013), which could explain the low levels currently observed in our mice sacrificed between 9 and 11am. It was our intention for the peak in blood alcohol to coincide with the time in which cage activity and therefore muscle loading was to be greatest (i.e., during the dark cycle).

In summary, moderate alcohol consumption did not alter muscle hypertrophy, protein synthesis, or the majority of mTORC1-related signaling events induced by 14 days of chronic muscle overload. These findings are relevant to patients with chronic alcohol-induced muscle disease as they indicate that a simple intervention such as daily weightlifting exercise could provide a sufficient anabolic stimulus to induce muscle (re)growth and potentially reverse or prevent further disease.

## Conflict of Interest

The authors have no conflicts of interest to declare.
